# Carbon nanorings with inserted acenes: breaking symmetry in excited state dynamics

**DOI:** 10.1038/srep31253

**Published:** 2016-08-10

**Authors:** R. Franklin-Mergarejo, D. Ondarse Alvarez, S. Tretiak, S. Fernandez-Alberti

**Affiliations:** 1 Universidad Nacional de Quilmes/CONICET, Roque Saenz Peña 352, B1876BXD Bernal, Argentina; 2 Theoretical Division, Center for Nonlinear Studies (CNLS), and Center for Integrated Nanotechnologies (CINT), Los Alamos National Laboratory, Los Alamos, NM 87545, USA

## Abstract

Conjugated cycloparaphenylene rings have unique electronic properties being the smallest segments of carbon nanotubes. Their conjugated backbones support delocalized electronic excitations, which dynamics is strongly influenced by cyclic geometry. Here we present a comparative theoretical study of the electronic and vibrational energy relaxation and redistribution in photoexcited cycloparaphenylene carbon nanorings with inserted naphthalene, anthracene, and tetracene units using non-adiabatic excited-state molecular dynamics simulations. Calculated excited state structures reflect modifications of optical selection rules and appearance of low-energy electronic states localized on the acenes due to gradual departure from a perfect circular symmetry. After photoexcitation, an ultrafast electronic energy relaxation to the lowest excited state is observed on the time scale of hundreds of femtoseconds in all molecules studied. Concomitantly, the efficiency of the exciton trapping in the acene raises when moving from naphthalene to anthracene and to tetracene, being negligible in naphthalene, and ~60% and 70% in anthracene and tetracene within the first 500 fs after photoexcitation. Observed photoinduced dynamics is further analyzed in details using induced molecular distortions, delocatization properties of participating electronic states and non-adiabatic coupling strengths. Our results provide a number of insights into design of cyclic molecular systems for electronic and light-harvesting applications.

Conjugated carbon nanorings comprise a wide variety of chemical compounds including different sizes of cycloparaphenylenes and related nanohoops^1^. They present unique combinations of strain, disorder, bending, and sterically hindrance that lead to particular variations of the conjugation extent within their molecular structure. Advances in the controlled synthesis of these compounds^2^,^3^,^4^,^5^,^6^,^7^,^8^,^9^,^10^ allow exploring the peculiar size-dependent trends of their linear and nonlinear optical properties^2^,^8^,^9^,^10^,^11^. Besides their fundamental interest, cycloparaphenylenes and other well-ordered macrocycles exhibit a myriad of technological applications, like organic field-effect transistors, and nonlinear optics^12^,^13^,^14^,^15^,^16^,^17^,^18^,^19^. The efforts in the organic synthesis and purification of bunches of these new synthetic nanohoops have led to exploring not only new molecules with ambitioned optoelectronic properties but also new environments and complexation reactions^20^,^21^,^22^,^23^,^24^,^25^,^26^,^27^. The detailed understanding, at the molecular level, and control of the electronic responses of these new organic nanostructures represent a significant contribution in the development of new nanoscale optoelectronic technologies.

Cycloparaphenylenes ([*n*]CPPs) are a class of carbon nanohoops which consist of *n* phenyl units linked at the *para* positions in a conjugated periodic chain^28^. They present unique physicochemical and electronic properties with the particular interest of being the smallest models of larger carbon nanotubes^29^,^30^,^31^,^32^,^33^. In this context, these materials not only have awaken the academic interest but also the development of applications in material science and technology. The constant new advances in the synthesis and characterization of CPPs and related nonohoops^2^,^3^,^4^,^5^,^6^,^7^,^8^,^9^,^10^ encourage new experimental and theoretical studies in order to achieve a detailed understanding of principal structural and dynamical features that furnish them with unique optoelectronic properties^2^,^4^,^28^,^34^,^35^,^36^,^37^,^38^,^39^.

CPPs are conjugated systems with radially oriented π-orbitals, creating a compromise between an optimal conjugation extent and steric hindrances between the comprising phenyl units^40^. As consequences, CPPs dihedral angles between the phenyl rings are non zero^41^,^42^ and π-orbital overlaps are less efficient, also due to structural distortions introduced in phenyl units. Exciton localization/delocalization within the nanoring is strongly affected by these features^11^,^43^. Besides, the insertion of other organic compounds between phenyls, like alkyl chains^44^ and acene units^45^,^46^,^47^, has a significant impact in their optical properties and it can, eventually, lead a gradual modulation of the length scale of exciton localization (self-trapping).

In the recent years, nanohoops combining different units^48^,^49^,^50^,^51^,^52^ have been successfully synthetized in an attempt to explore new features that connect these models with single-walled carbon nanotubes (SWCNTs)^1^,^35^,^42^,^53^,^54^,^55^,^56^,^57^,^58^,^59^,^60^,^61^. CPPs with inserted acene units, like naphthalene (CPPN), anthracene (CPPA), and tetracene (CPPT), represent the shortest chiral SWCNT structures. The acene units are inserted with 2,6-linkage, introducing a helical chirality. The racemization of the resulting chiral carbon nanonorings occurs via the rotation of the inserted acene units around the two C-C bonds that connect it to the adjacent phenyl units. According to studies performed by Itami *et al*.^47^ the calculated racemization barriers implicates that this compounds undergo rapid racemization at ambient temperature.

In the present paper, we explore modifications of optical/electronic properties and photoinduced dynamics due to the insertion of an acene unit that breaks the circular symmetry of CPP. In unmodified CPPs, all electronic states are fully delocalized across the entire molecule^11^. The acene units gradually break this symmetry, introduce localized electronic states and significantly alter optical selection rules^62^. For example, the lowest excited state being optically forbidden in ideal structures, gains a significant transition dipole moments oriented along acene’s short axis. Such transformations of electronic structure has characteristic footprints in photoexcited non-radiative relaxation as shown with our non-adiabatic excited-state molecular dynamics (NA-ESMD)^63^,^64^ simulations. Calculated timescales are analyzed in detail using electronic transition density evolution during the internal conversion processes, showing gradual spatial localization of excited state wavefunctions. Accompanying vibrational energy relaxation and redistribution are rationalized by examining bond-length alternation parameters and torsional motions.

## Results and Discussion

### Ground state structures and optical absorption

**Figure 1:**
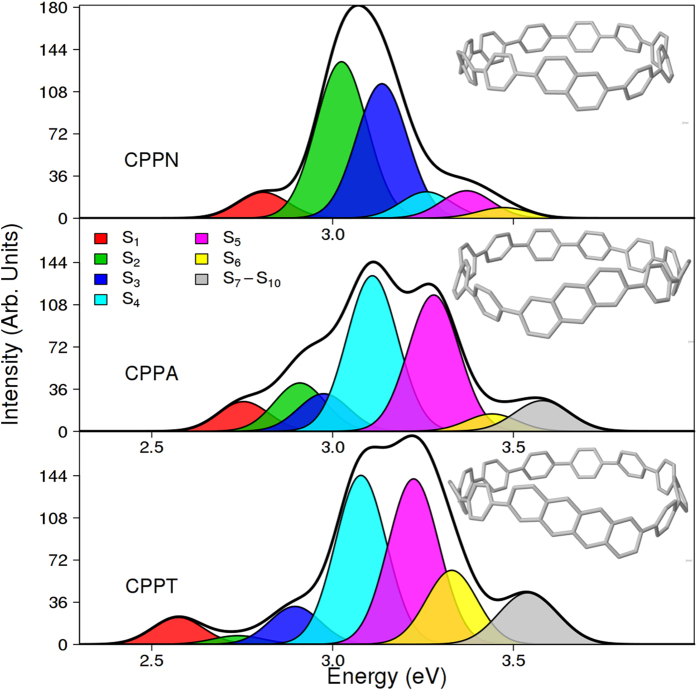
Simulated absorption spectra of [10]CPPN, [10]CPPA, and [10]CPPT with separated contributions of different excited states. Molecular structures are shown in the respective insets. Absorption profiles were obtained using an effective Gaussian full width half maximum of 0.01 eV.

We have modeled three [10]CPPs molecules with inserted naphthalene (CPPN), anthracene (CPPA), and tetracene (CPPT) units. The chemical structures of these compounds are depicted in Fig. 1 (insets). The size of nanohoops have been chosen considering that ten phenyl rings represent a balance competition between maintaining the aromaticity of the individual benzene rings versus minimizing the strain energy along the backbone chain^11^. Despite that [10]CPP and [10]CPPN have been shown to present close lowest excitation energies, the S_1_ oscillator strength for [10]CPPN results substantially higher than [10]CPP^45^. The analysis of ground-state conformational sampling at 300 K indicates that all benzene and acene units are alternatively twisted. The average dihedral angles between neighboring phenyls are 35.2° ± 14.6°, 35.7° ± 14.6°, and 36.8° ± 15.8°, for CPPN, CPPA, and CPPT, respectively. Previous studies performed on CPP molecules^2^,^4^,^34^ have reported an increase on the average values of dihedral angles with the size of the ring. Therefore, our results are a consequence of a slight increase of the nanoring size due to acene units. Moreover, the average dihedral angles at the acene-phenyl hetero-junctions are 34.3° ± 14.6°, 33.9° ± 14.7°, and 32.9° ± 14.4°, for CPPN, CPPA, and CPPT, respectively. As it has been previously pointed out for the CPP nanorings^2^,^4^,^34^, conformational disorder induced by temperature leads to wider dihedral angle distributions, reducing the extent of π-conjugation.

Calculated absorption spectra are shown in Fig. 1, where the colored profiles indicate individual contributions of different excited states. Compared to unsubstituted CPPs^11^, and as it has been previously pointed out^45^, the excitation to the lowest S_1_ excited-state is no longer forbidden by gaining substantial oscillator strengths in all structures considered. This is a consequence of the geometric symmetry-breaking generated by the insertion of the acene units. The S_1_ state shows a peak that is displaced to lower energies, while increasing the size of the acene unit (at 2.81 eV, 2.75 eV, and 2.57 eV for CPPN, CPPA, and CPPT, respectively). The S_1_ oscillator strength is slightly higher in CPPA compared to that in CPPN, and CPPT. According to Wong *et al*.^45^, this is a consequence of the relative alignment of the orbital energies between the different acenes and the phenylene backbone. The overall width of the absorption profiles grows with an increase of the acene size reflecting a large number of optically active excited states (we recall that the entire uv-vis spectra of all pristine [*n*]CPPs^11^ are essentially defined by solely S_2_ and S_3_ states).

**Figure 2:**
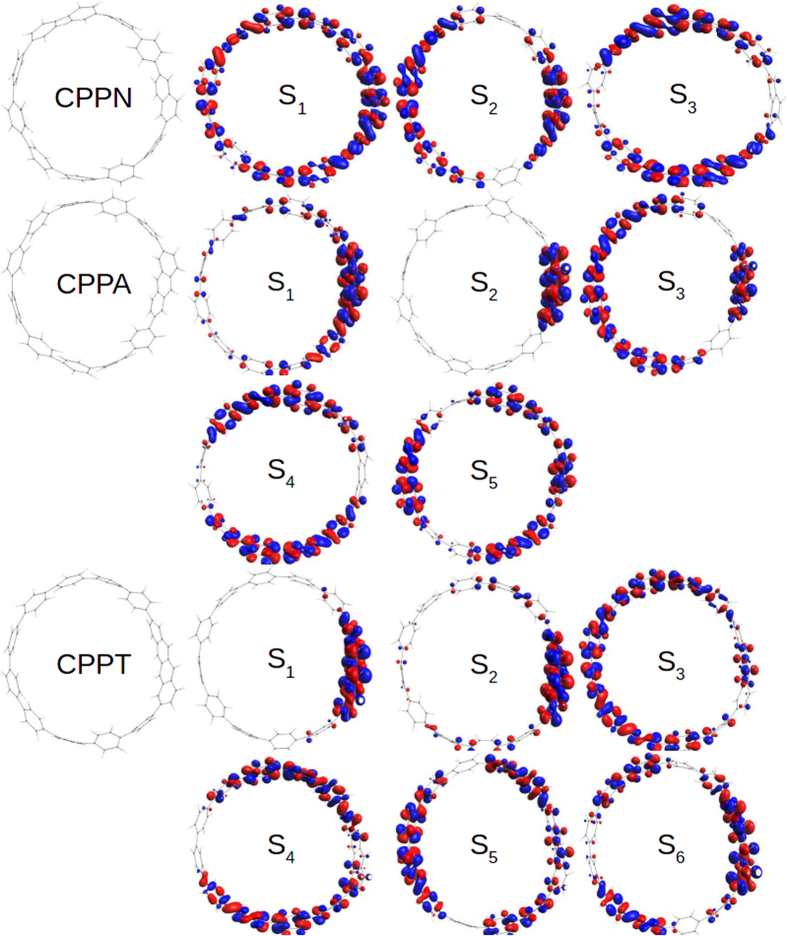
Spatial distribution of transition densities of the essential electronic excited states in CPPN, CPPA, and CPPT, calculated at their corresponding ground-state energy minima.

We further examine spatial distributions of transition densities corresponding to the essential electronic excited states in CPPN, CPPA, and CPPT shown in Fig. 2. Overall, we observe more pronounced symmetry breaking of the ring leading to state localization for larger acenes. In particular, the S_1_ transition density is highly delocalized around the entire CPPN structure, with a slight preference to the naphthalene unit. In contrast, the S_1_ state becomes localized on a semicircle including the anthracene in CPPA, and it is highly localized on the tetracene in CPPT. These asymmetric distributions of the S_1_ transition densities explain the growth of the transition dipole in these compounds relative to [*n*]CPPs^11^ (for a more direct comparison with [10]CPP, see SI of ref. 11). Here, CPPA case maximizes the cooperative contributions of the acene and the phenyl ring backbone to the final transition dipole moment^45^. As it has been previously reported for [*n*]CPPs^11^, transition density of the higher-energy S_2_ and S_3_ excited-states has two nodes on the opposite side of the ring and, therefore, a constructive superposition of individual phenyl dipoles is assured. The same case holds for CPPN (Fig. 2) underlining the similarity to [*n*]CPPs, however, the degeneracy of S_2_ and S_3_ states is lifted. As it can be observed in Fig. 1, these states have large oscillator strengths, making CPPN an excellent optical absorber. In the case of CPPA and CPPT, the S_2_ states are strongly localized on the acene unit and, subsequently, have relatively small oscillation strength. Among the higher-energy excited states, S_4_ and S_5_ retain the characteristic nodal structure and provide the strongest contributions to the CPPA, and CPPT absorption spectra (Fig. 1).

### Excited state dynamics and non-radiative relaxation

**Figure 3:**
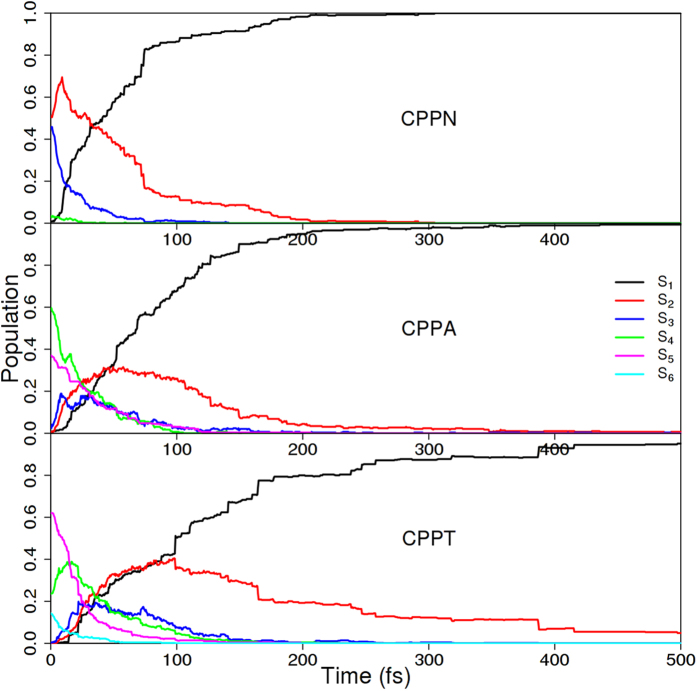
Population of each electronic state calculated from the number of trajectories in a state at a given time after excitation for three molecular compounds.

After examination of electronic states, we turn to the results of the NA-ESMD simulations. We start from the equilibrated ground-state configurations by instantaneously promoting the system to excited state near the maximum of the absorption spectra (Fig. 1), that is, 3.07 eV (403 nm), 3.17 eV (391 nm), and 3.12 eV (386 nm) for [10]CPPN, [10]CPPA, and [10]CPPT molecules, respectively. The number of electronic excited states included in the simulations varied according to their contributions to the absorption spectra, being 6, 10, and 10 for CPPN, CPPA, and CPPT, respectively. The time evolution of the populations on each electronic excited state after photoexcitation is shown in Fig. 3. The laser excitation wavelength for each molecular system, corresponds mainly to S_2_/S_3_ in CPPN, and S_4_/S_5_ in CPPA and CPPT. After photoexcitation, an ultrafast electronic energy relaxation to the lowest excited state (S_1_) is observed on the time scale of hundreds of femtoseconds. The relaxation process for a trajectory ensemble generally follows a sequential mechanism *S*_*n*_  → … → *S*_*2*_ → *S*_*1*_. The resulting total relaxation rate is getting slower with an increase of the acene size. The build-up of the S_1_ state populations are well fitted to a single exponential function *f*(*t*) = 1 − *A*_0_*e*^−*t*/*τ*^, where *τ* is an effective relaxation time with values of 54 fs, 64 fs, and 118 fs for CPPN, CPPA, and CPPT systems, respectively.

**Figure 4:**
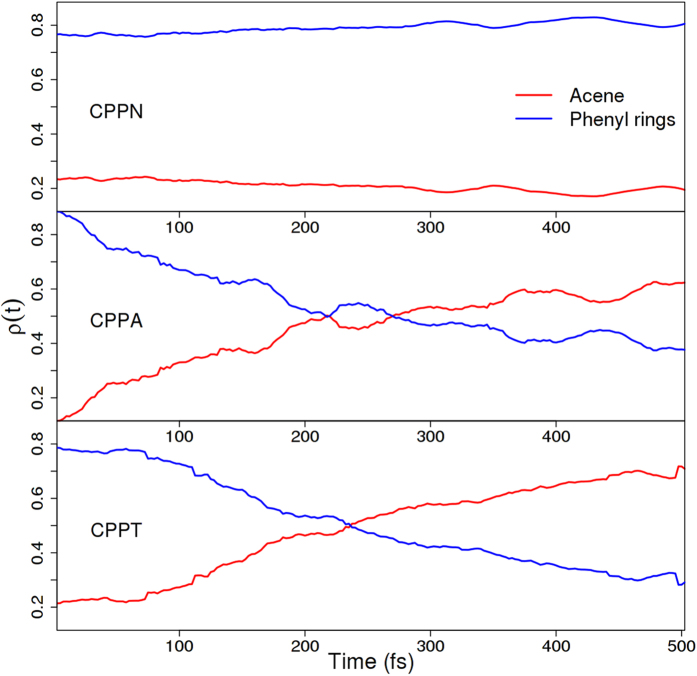
Time-dependence of the average fraction of transition density localized on the acene (red) and the phenyl rings (blue).

The intramolecular electronic energy redistribution that takes place during internal conversion has been monitored through 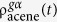, being the average fraction of electronic transition density localized on the acene units. The time-evolution of 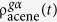 for three molecules is shown in Fig. 4. Essentially, 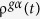 remains highly delocalized across the entire CPPN structure (about 80%) with no significant variations throughout the NA-ESMD simulations. This results from the delocalization of transition densities of excited states in CPPN, shown in Fig. 2. In contrast, 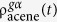 significantly varies in CPPA and CPPT. In both cases, the initial excitation is delocalized around the phenyl rings (about 90%) with only a minor fraction located on the anthracene and tetracene. After photoexcitation, however, the exciton undergoes an ultrafast migration to the acenes, which trap on average over 60% of the wavefunction toward the end of non-radiative relaxation. This process is more effective in CPPT compared to CPPA. In CPPT, the exciton finishes almost entirely localized on the tetracene. The rise of 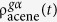 are well fitted to a single exponential function *f*(*t*) = *A*_1_ − *A*_0_*e*^−*t*/*τ*^, where *A*_1_ is the asymptotic limit with values of 0.66 and 1 for CPPA, and CPPT systems, respectively. Following previous association of the CPP exciton with a “quasiparticle” evolving on a circular geometry (see ref. 11), here we can relate CPPs with inserted acene units to a “quasiparticle on a circle with a finite potential well”. Tetracene represents a deeper well than anthracene, favoring the localization of the lowest excited-states to the acenes and, therefore, the final exciton trapping to an effective defect state.

### Analysis of coupled electron-vibrational dynamics

**Figure 5:**
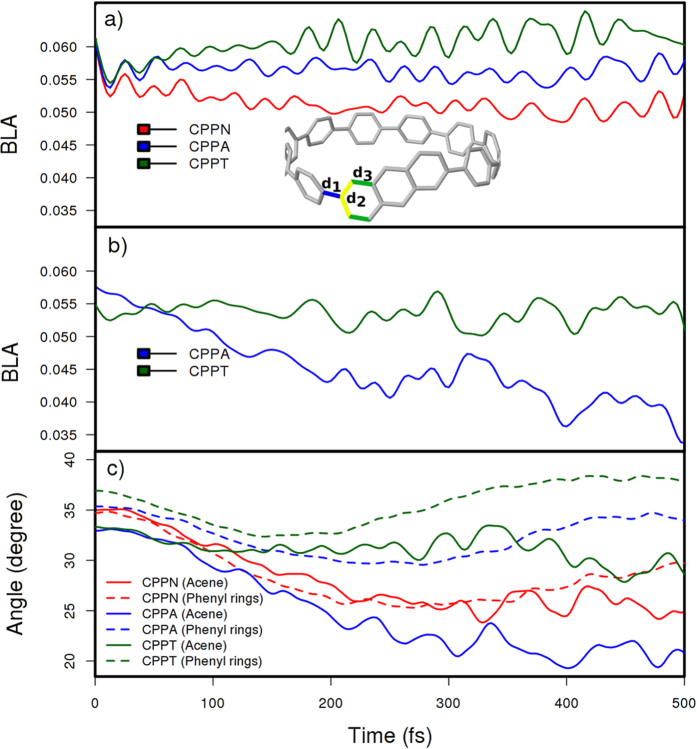
Time evolution of the average bond length alternations (BLA) (**a**) between neighboring phenyls and (**b**) between the acene unit and its neighboring phenyls. (**c**) Time evolution of the average dihedral angles between the neighboring phenyls (dashed) and between the acene and its neighboring phenyls (solid).

In order to analyze the structural distortions during the intramolecular energy redistribution and vibrational motions receiving electronic energy after photoexcitation, we have monitored the torsions (dihedral angles) and bond length alternations (BLA) between the neighboring phenyls and between the acene unit and its neighboring phenyls. The BLA parameter is defined as a difference between single and double bond lengths


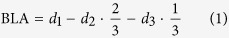


where *d*_1_,*d*_2_ and, *d*_3_ are labeled in Fig. 5. Generally, small values of dihedral angles and BLA reflect more effective π-conjugation across the backbone. The time-evolution of the average values of BLA is depicted in Fig. 5. After photoexcitation, all three molecular systems (CPPN, CPPA, and CPPT) experience an initial ultrafast reduction of BLA between the neighboring phenyls (Fig. 5a). In the case of CPPN, this reduction persists throughout the entire simulation time. This reflects the exciton delocalization across the entire nanoring previously shown in Figs 2 and 4, which distorts molecular geometry to create a better conjugation path^65^. In contrast, the average BLA between phenyls in CPPT recovers its initial values associated with the ground state geometry. This manifests excitation trapping on the tetracene as a result of the electronic relaxation, as shown in Fig. 4. Finally, an intermediate behavior is observed for CPPA, presenting a partial recovery of the BLA initial values associated to a partial exciton delocalization between the phenyl and anthracene units. Figure 5b shows time-dependence of the BLA at the acene-phenylene heterojunction for [10]CPPA, and [10]CPPT systems presenting structural footprints of exciton localization on the acenes. We observed that, while the anthracene-phenylene junction bears a significant reduction of its initial values, this is not the case for tetracene-phenylene one. These different behaviors are a consequence of the different extent of the exciton localization through the heterojunctions. That is, π-conjugation tends to reduce the values of BLA at the acene-phenylene heterojunction. Small values of BLA between the acene and its neighbouring phenyls are subsequently associated with an extent of the π-conjugation, and therefore an exciton delocalization, throughout this heterojunction. As shown in Fig. 4, the final transition density in the CPPA is partially delocalized across the nonoloop, which includes the anthracene unit. In contrast, the final exciton in the CPPT is mostly concentrated on the tetracene unit. These vibrational energy redistributions, concomitant with the electronic energy relaxation, are also reflected in changes of the average dihedral angles between neighboring phenyls and between the acene – phenyl junctions as plotted in Fig. 5c. The reduction of dihedral angles after photoexcitation follows equivalent trend observed for the BLA, that is, a larger effect is noticed when moving from CPPT to CPPA and to CPPN. The dihedral angles at the acene-phenyl junction behave differently compared to phenyl-phenyl torsions. While both types of dihedral angles are very similar behavior in CPPN, anthracene-phenyl torsions in CPPA have the largest decrease. These results, in conjunction with the corresponding BLA reduction in Fig. 5b, indicate CPPA as the molecular system where the medium-depth defect partially traps the excitonic wavefunction, while preserving acene-nanohoop electronic delocalization.

**Figure 6:**
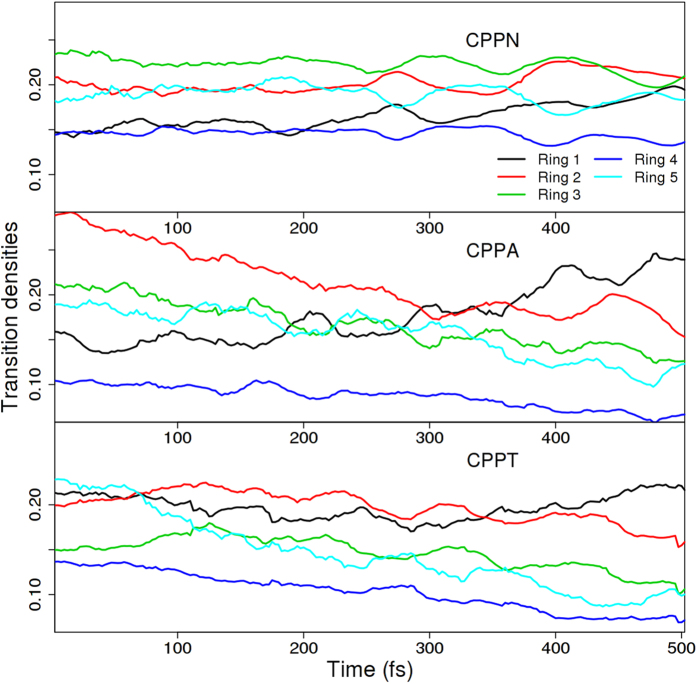
Time evolution of the average fraction of transition density on each of the phenyls in the nanohoop. The rings are enumerated according to their proximity to the acene. The values are normalized with respect to the fraction of transition density on the entire nanoring excluding the contribution of the acene.

A quantitative measure of excitonic intramolecular redistribution within the nanohoop is provided by the analysis of the fraction of transition density on each of its phelyl unit as shown in Fig. 6. Here the phenyl rings are enumerated according to their proximity to the acene unit and plotted values are normalized with respect to the fraction of transition density on the nanoring excluding the acene unit. Only a slight increase in the localization on the phenyls closest to the acene is observed in CPPN. The fraction of transition density on the other rings is seemingly not affected by the electronic relaxation process. In the case of CPPA, phenyls closest to the anthracene experience larger increase of their transition density fraction with time compared to that in CPPN. In CPPT, we observe an average decrease of excitation density across all phenyls except the ones closest to the tetracene, which seemingly retain their values throughout the simulations.

**Figure 7:**
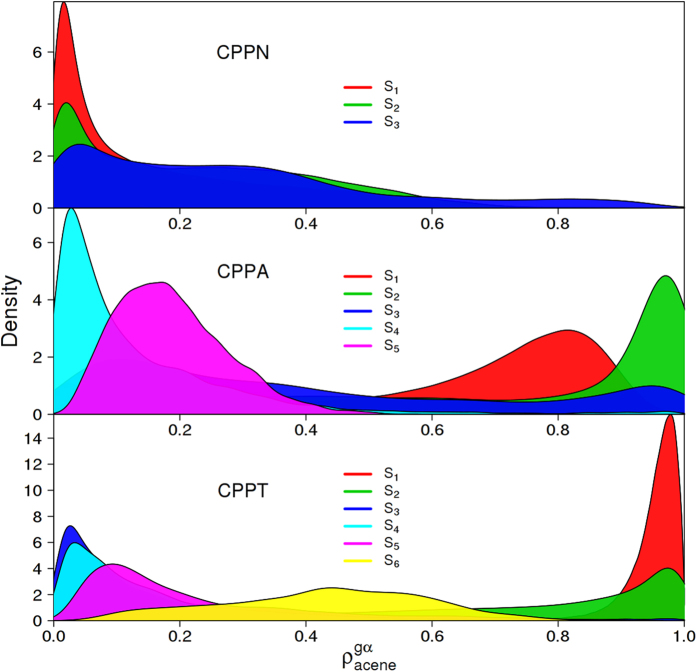
Histograms for the fraction of transition density on the acene integrated for the trajectory ensemble while system is evolving on the S_i_ state during the NA-ESMD simulations.

The electronic energy relaxation that takes place after photoexcitation involves the participation of several intermediate excited states that, according to their lifetimes, act in the exciton delocalization/localization and intra-ring migration. In order to analyze this feature, Fig. 7 displays histograms of the fraction of electronic transition density localized on the acene units, 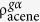, while nuclei are moving on each S_α_ excited-state throughout the NA-ESMD simulations. In that way, we can identify the role of each state during the exciton delocalization->localization process. CPPN excited-states are rarely localized on naphthalene with an exception of S_3_ state. In contrast, much broader variety of contributions from excited-states results in the exciton localization in CPPA and CPPT. While high-energy excited states are mainly delocalized on the nanohoop, low-energy excited states reinforce trapping the exciton on the acene. In both cases, S_1_ and S_2_ provide the strongest localization. Nevertheless, both states differentiate in their spatial localization in CPPA and CPPT, concomitant with transition density plots in Fig. 2.

## Conclusions

Our computational study explored electronic structure and non-radiative relaxation (internal conversion) in a series of cycloparaphenylenes (CPP) with inserted naphthalene (CPPN), anthracene (CPPA), and tetracene (CPPT) units, breaking the cyclic symmetry. Examination of the wavefunctions of electronically excited states has shown that presence of the acenes on a circle can be considered as an introduction of effective defects sites, which can strongly or weakly mixed with the delocalized excitonic bands. Naphthalene can be considered as shallow defect barely breaking the symmetry. Anthracene introduces deeper energy states, which are still well mixed with delocalized excitations. Finally, tetracene insertion leads to an appearance of fully localized states on the defect. Within this context, our findings suggest anthracene defect as a more malleable defect for future design of nanorings envisaging light-harvesting applications, compared to the weak effect of naphthalene and the strong effect reported for tetracene. Introduction of adequate substituents in the nanohoop can tilt the localization/delocalization balance in the desired direction to achieve specific synthetization points on the structure acting as sinks for electronic energy fluxes.

The resulting optical absorption spectra significantly broaden due to modification of selection rules and participation of a larger number of excited states along the series CPPN, CPPA and CPPT, reflecting stronger symmetry break. For example, unlike unmodified CPPs, the excitation to the lowest S_1_ excited-state becomes optically allowed in all three systems.

The NA-ESMD simulations further deliver detailed information on the electronic and vibrational energy relaxation and redistribution at room temperature in photoexcited molecules. In all three systems, an ultrafast electronic energy relaxation to the lowest excited state is observed on the time scale of hundreds of femtoseconds, following a sequential *S*_*n*_ → … → *S*_*2*_ → *S*_*1*_ mechanism. The relaxation process to the lowest S_1_ excited state slows down with an increase of the acene size. In parallel, more effective trapping (localization) of the electronic excitation on the defect site is observed toward the end of an internal conversion process.

The time-dependent and spatially resolved electronic transition density distribution during the internal conversion processes has been monitored, focusing on gradual changes of its localization on the acene subunits. Exciton localization on the acenes has been shown to be more effective as the size of the acene unit increases. While the transition density remains essentially delocalized around the entire nanohoop for CPPN during the entire dynamics, it experiences an ultrafast self-trapping on the acene units for CPPA and CPPT. This process is more effective in CPPT than in CPPA, where the intramolecular energy redistribution after photoexcitation implies a gradual migration of the exciton in the direction to the acene trap, involving most of phenyls in the nanohoop. Excitation and relaxation of vibrational degrees of freedom, concomitant to the electronic energy evolution, has been analyzed through the structural distortions induced by the photoinduced process. Variations in both the bond length alternations and dihedral angles are consistent with the intramolecular exciton migration during the electronic relaxation.

Overall, our results elucidate the dynamical interplay between exciton migration/trapping and structural symmetry-breaking in conjugated nonohoops, which have significant effects on the optical and electronic properties of these compounds. Detailed analysis expose intrinsic connections of intramolecular exciton dynamics, energy transfer and structural rearrangements in the electronic energy relaxation process following optical excitation. Light-harvesting processes within these nanohoops can be visualized as complex exciton delocalization-localization processes controlled by competition between intermediate electronic excited-states with different optical properties and lifetimes, and the concomitant nuclear responses allowing exciton redistribution. We show that bond lengths and dihedral angles vary accordingly within the electronic relaxation time-scale. Therefore, any substituents or defects introduced in the nanohoops modifying these structural features can be proposed as strategies to manipulate the process. These findings highlight an excellent value of synthetic fully conjugated nanorings as unique class of organic semiconductors for electronic and the light-harvesting applications.

## Methods

### The NA-ESMD overview

The NA-ESMD framework^64^,^63^ is a direct non-adiabatic molecular dynamics method that combines the Fewest Switches Surface Hopping (FSSH) algorithm^66^,^67^ with “on the fly” analytical calculations of excited-state energies^68^,^69^,^70^, gradients^71^,^72^, and non-adiabatic coupling terms^63^,^73^,^74^,^75^. Briefly, the FSSH is a mixed quantum classical approach in which nuclei are treated classically and are propagated according to forces from a single adiabatic excited state at any given time. Transitions from one excited state to another occur stochasticically based on the nonadiabatic coupling strengths. Meanwhile, electrons have a quantum mechanical description related to excited state energies, gradients, and non-adiabatic coupling terms. The electronic excited states are calculated with Collective Electronic Oscillator (CEO) method^76^,^77^,^78^ using the Configuration Interaction Singles (CIS) formalism implemented with the semiempirical Austin model 1 (AM1) Hamiltonian^79^, providing efficient evaluations of excited state energies, gradients and non-adiabatic couplings. AM1 Hamiltonian includes all valence electrons and use truncated two-electron integrals approximate form. In order to validate our level of theory, AM1/CIS vertical excitation energies for S_n_ states (n = 1–6) have been compared with time-dependent DFT calculations at the B3LYP level using the 6–31G(d,p) basis set, previously reported by Wong *et al*.^45^. Results are shown in Table S1. The agreement between the AM1 and the TDDFT results is quantitative for transition energies of multiple essential states defining electronic dynamics in this molecular family. The NA-ESMD has been extensively used to simulate internal conversion processes involving manifold of coupled electronic excited-states in a broad variety of extended conjugated molecules^80^,^81^,^82^,^83^, including [n]CPPs^84^.

During the NA-ESMD simulations, the time-dependent localization of the electronic transition density is calculated using its diagonal elements (ρ^*gα*^)_*nn*_ (index *n* refers to atomic orbital (AO) basis functions) that represent the changes in the distribution of the electronic density induced by photoexcitation from the ground state *g* to an excited electronic *α* state^85^. The fraction of electronic transition density localized on the acene units of CPPN, CPPA, and CPPT is evaluated as:


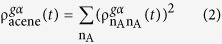


where the index A runs over all atoms localized in the acene unit.

### Computational details

One nanosecond ground state molecular dynamics simulations was performed for initial equilibration of all molecular structures studied. We use Langevin thermostat with temperature T = 300 K, a friction coefficient γ = 2.0 ps^−1^ and time step Δ*t* = 0.5 fs. The ground state trajectory was used to collect sets of initial configurations for the subsequent NA-ESMD simulations. The NA-ESMD simulations have started from these initial configurations by instantaneously promoting the system to an initial excited state α with the frequency 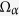, selected according to a Frank-Condon window defined as 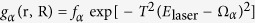. *f*_*α*_ represents the normalized oscillator strength for the α state, and *E*_laser_ represents the energy of a laser pulse centered at the maximum of the absorption spectra of a given molecule. The excitation energy width is given by the transform-limited relation of a Gaussian pulse with a FWHM of 100 fs, giving a value of T^2^ = 42.5 fs. Using 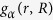, the initial excited state for each structure was determined. Having chosen the initial excited state, a NAESMD simulation (trajectory) was carried out for every structure during 500 fs using a Langevin thermostat at T = 300 K and γ = 2.0 ps^−1^. The influence of the laser field, after the excited state has been populated, has not been considered during the NA-ESMD. 400 NA-ESMD trajectories were started from these initial configurations. In agreement with previous numerical tests performed on NA-ESMD, 400 trajectories is found to be sufficient to achieve statistical convergence^86^. A classical time step of 0.1 fs has been used for nuclei propagation and a quantum time step of 0.025 fs has been used to propagate the electronic degrees of freedom. Empirical corrections were introduced to account for electronic decoherence^87^. Trivial unavoided crossings were diagnosed by tracking the identities of states^88^. More details concerning the NA-ESMD implementation and parameters can be found elsewhere^63^,^86^,^89^.

## Additional Information

**How to cite this article**: Franklin-Mergarejo, R. *et al*. Carbon nanorings with inserted acenes: breaking symmetry in excited state dynamics. *Sci. Rep*. **6**, 31253; doi: 10.1038/srep31253 (2016).

## Supplementary Material

Supplementary Information

Supplementary Movie

Supplementary Movie

Supplementary Movie
