# Supplementary material for: Carbon nanorings with inserted acenes: breaking symmetry in excited state dynamics
Source: Sci Rep. 2016 Aug 10;6:31253. doi: 10.1038/srep31253 (PMC4978956; doi:10.1038/srep31253)
Supplement: Supplementary Information [file srep31253-s1.pdf]

Supplementary Information

Carbon nanorings with inserted acenes: breaking symmetry in excited state dynamics

R. Franklin-Mergarejo[1], D. Ondarse Alvarez[1], S. Tretiak[2*], S. Fernandez-Alberti[1*]

[1]Universidad Nacional de Quilmes/CONICET, Roque Saenz Peña 352, B1876BXD Bernal, Argentina

[2]Theoretical Division, Center for Nonlinear Studies (CNLS), and Center for Integrated Nanotechnologies (CINT), Los Alamos National Laboratory, Los Alamos, NM 87545, USA.

Table S1. Comparison of vertical excitation energies (eV) for S_n (n=1-6) calculated at the AM1/CIS level and TDDFT (B3LYP/6-31G*) for an AM1-optimized structure.

		S_1	S_2	S_3	S_4	S_5	S_6
**CPPN**	AM1/CIS	2.91	3.11	3.19	3.25	3.43	3.52
	TDDFT	3.09	3.39	3.39	3.41	3.65	3.79
**CPPA**	AM1/CIS	2.79	2.96	2.99	3.13	3.29	3.44
	TDDFT	2.79	3.15	3.18	3.26	3.36	3.37
**CPPT**	AM1/CIS	2.60	2.75	2.96	3.12	3.24	3.32
	TDDFT	2.33	2.89	2.96	3.02	3.05	3.17

CPPN.mov, CPPA.mov, and CPPT.mov depict the evolution in time of the spatial distribution of transition density throughout a typical NA-ESMD trajectory for CPPN, CPPA, and CPPT molecules respectively. The molecular systems are oriented with the acene units localized at the botton.
